# Metformin Alters mRNA Expression of *FOXP3*, *RORC*, and *TBX21* and Modulates Gut Microbiota in COVID-19 Patients with Type 2 Diabetes

**DOI:** 10.3390/v16020281

**Published:** 2024-02-11

**Authors:** Pavlo Petakh, Iryna Kamyshna, Valentyn Oksenych, Oleksandr Kamyshnyi

**Affiliations:** 1 Department of Biochemistry and Pharmacology, Uzhhorod National University, 88000 Uzhhorod, Ukraine; 2 Department of Microbiology, Virology, and Immunology, I. Horbachevsky Ternopil National Medical University, 46001 Ternopil, Ukraine; 3 Department of Medical Rehabilitation, I. Horbachevsky Ternopil National Medical University, 46001 Ternopil, Ukraine; kamyshna_ii@tdmu.edu.ua; 4 Broegelmann Research Laboratory, Department of Clinical Science, University of Bergen, 5020 Bergen, Norway

**Keywords:** COVID-19, metformin, Treg, T cell, Th17, F/B ratio, diabetes, SARS-CoV-2

## Abstract

COVID-19 remains a significant global concern, particularly for individuals with type 2 diabetes who face an elevated risk of hospitalization and mortality. Metformin, a primary treatment for type 2 diabetes, demonstrates promising pleiotropic properties that may substantially mitigate disease severity and expedite recovery. Our study of the gut microbiota and the mRNA expression of pro-inflammatory and anti-inflammatory T-lymphocyte subpopulations showed that metformin increases bacterial diversity while modulating gene expression related to T-lymphocytes. This study found that people who did not take metformin had a downregulated expression of *FOXP3* by 6.62-fold, upregulated expression of *RORC* by 29.0-fold, and upregulated *TBX21* by 1.78-fold, compared to the control group. On the other hand, metformin patients showed a 1.96-fold upregulation in *FOXP3* expression compared to the control group, along with a 1.84-fold downregulation in *RORC* expression and an 11.4-fold downregulation in *TBX21* expression. Additionally, we found a correlation with gut microbiota (F/B ratio and alpha-diversity index) and pro-inflammatory biomarkers. This novel observation of metformin’s impact on T-cells and gut microbiota opens new horizons for further exploration through clinical trials to validate and confirm our data. The potential of metformin to modulate immune responses and enhance gut microbiota diversity suggests a promising avenue for therapeutic interventions in individuals with type 2 diabetes facing an increased risk of severe outcomes from COVID-19.

## 1. Introduction

Over four years have elapsed since the emergence of the new Severe Acute Respiratory Syndrome Coronavirus 2 (SARS-CoV-2) virus, impacting over 700 million people globally [1,2]. Despite the World Health Organization officially ending the pandemic status in 2023, the emergence of new variants such as Pirola and Eris continues to pose a serious threat [3]. Numerous studies have identified type 2 diabetes (T2D) as a significant risk factor for severe Coronavirus disease 2019 (COVID-19), often resulting in hospitalization or death [4]. Elevated levels of hyperglycemia and glycemic fluctuations may adversely affect COVID-19 outcomes, with some studies suggesting that insulin treatment could contribute to increased mortality in COVID-19 patients with T2D [5,6]. Additionally, T2D may impair nasal immunity, increasing the risk of hyposmia in individuals with mild COVID-19 pneumonia [7].

Metformin, a widely used oral antidiabetic medication, exhibits pleiotropic effects beyond glycemic control [8,9]. One potential mechanism for its beneficial effects is its anti-inflammatory properties [10,11,12,13]. Metformin may influence the immunometabolism of lymphocytes, impacting the differentiation and balance between pro-inflammatory T helper 1 (Th1) and T helper 17 (Th17) cells, as well as anti-inflammatory regulatory T cells (Treg) [14]. This influence has the potential to reduce inflammation and the risk of cytokine storms in COVID-19 patients with T2D [15,16,17,18]. The mechanism is achieved through the activation of AMP-activated protein kinase (AMPK), the master sensor and regulator of cellular energy metabolism in mammals, which inhibits T cell differentiation by suppressing the mammalian target of rapamycin (mTOR) and Glucose transporter 1 (GLUT1) [14,16,17,19]. This inhibition leads to a decrease in glucose uptake and glycolysis, limiting the energy supply available for T cell activation and proliferation.

In addition to AMPK, there are three key genes involved in the transcriptional regulation of Treg, Th1, and Th17 differentiation. These genes include *FOXP3*, *TBX21*, and *RORC*. FOXP3 (*FOXP3* gene) is a transcription factor essential for the development and function of Tregs, which play a crucial role in maintaining immune homeostasis and suppressing excessive immune responses [20]. T-be (*TBX21* gene) t is a transcription factor that promotes the differentiation of Th1 cells, which are involved in cell-mediated immune responses and the clearance of intracellular pathogens [21]. RORγt (*RORC* gene) is a transcription factor that drives the differentiation of Th17 cells, which are important for the defense against extracellular pathogens and the regulation of autoimmune responses [22]. Together, these three genes play critical roles in the regulation of immune responses and maintaining the balance between immune activation and tolerance. 

The balance between Th1, Th2, Th17, and Treg cells is crucial for maintaining immune homeostasis and preventing excessive inflammation. The dysregulation of these cells has been implicated in various autoimmune diseases and chronic inflammatory conditions. In the context of COVID-19, an imbalance in the activation of these immune cell subsets can contribute to the severity of the disease. For example, an exaggerated Th1 response may lead to excessive inflammation and tissue damage, while an impaired Th17 response could compromise the clearance of extracellular pathogens such as the SARS-CoV-2 virus [23]. Additionally, a dysregulated Th2 response may result in a reduced ability to produce antibodies and mount an effective immune response against the virus. On the other hand, regulatory Tregs are essential for maintaining immune tolerance and preventing excessive immune activation [24]. Therefore, understanding and modulating the balance of Th1, Th2, and Th17 cells could be a key factor in developing therapeutic strategies for COVID-19, with the aim of reducing inflammation and promoting a more balanced immune response. 

The composition of the gut microbiota undergoes alterations in individuals with both T2D and COVID-19. The gut microbiota, including bacteria and their metabolites, plays a role in influencing inflammation in the lungs through the bidirectional communication pathway known as the gut–lung axis [25]. An essential consideration is understanding how the most common oral hypoglycemic agent affects gut microbiota and inflammatory markers in patients with both COVID-19 and T2D. The most common oral hypoglycemic agent, metformin, has been found to have beneficial effects on gut microbiota and inflammatory markers in patients with both COVID-19 and T2D. Studies have shown that metformin can promote the growth of beneficial bacteria, such as *Akkermansia muciniphila*, and reduce the levels of pro-inflammatory cytokines in the gut [26]. This dual effect may contribute to improved outcomes in COVID-19 patients with T2D, as it could potentially reduce lung and systemic inflammation.

This study aims to investigate how the commonly used oral hypoglycemic agent metformin influences gut microbiota and inflammatory markers in individuals with both COVID-19 and T2D.

## 2. Materials and Methods

### 2.1. Sample Collection

**Figure 1:**
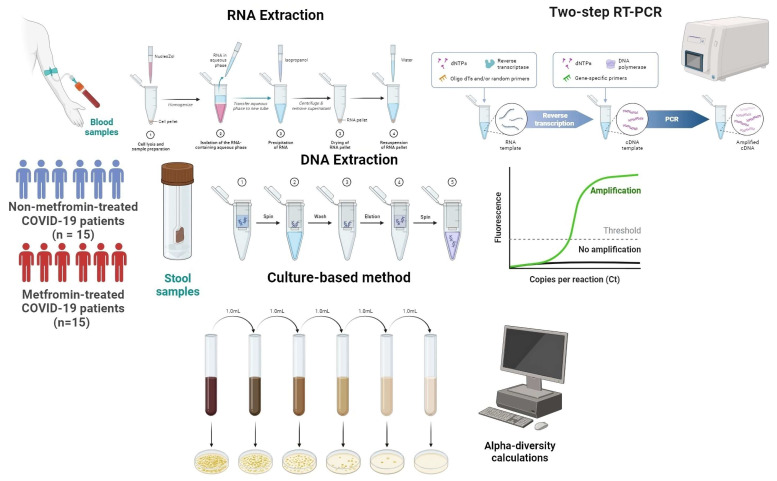
Study flow diagram with information on the methods used. We used two types of biological material from patients with COVID-19: stool samples and blood. The microbiota was studied using culture-based method (to calculate alpha-diversity indices) and molecular genetic testing (to calculate the Firmicutes/Bacteroidetes (F/B) ratio). The determination of the relative normalized expression of the studied genes was carried out by the method of polymerase chain reaction with reverse transcription.

Blood samples were collected from two groups of patients: first, COVID-19 patients with T2D and metformin treatment (metformin-treated group); and second, COVID-19 patients with T2D without metformin treatment (non-metformin-treated group). The samples were collected at Transcarpathian Regional Infectious Hospital. All participants provided informed consent by signing a statement. This study adhered to the principles of the Declaration of Helsinki and received approval from the Ethics Committee of I. Horbachevsky Ternopil National Medical University (protocol code 74, dated 1 September 2023). The blood was collected in EDTA tubes and stored at −80 °C until further use (Figure 1).

All participants tested positive for SARS-CoV-2, and patients with type 2 diabetes (T2D) were diagnosed based on the criteria set by the American Diabetes Association. Inclusion criteria for all groups were as follows: age between 25 and 75 years, no history of other chronic diseases, and no use of antibiotics or probiotics in the past 3 months. Exclusion criteria included pregnancy, lactation, a history of inflammatory bowel disease, and other gastrointestinal disorders. Participants were requested to provide a single stool and blood specimen concurrently. Patients taking metformin were administered a dose of 1000–1500 mg per day for at least 3 months before admission.

### 2.2. Clinical Data

The medical records of patients were reviewed to obtain clinical data such as NLR (Neutrophil-to-Lymphocyte ratio), CRP (C-Reactive Protein), Procalcitonin (PCT), and monocytes.

### 2.3. Gene Expression Analysis

#### 2.3.1. RNA Extraction and cDNA Synthesis

Total RNA was extracted from the collected blood samples using a standard protocol using NucleoZOL (740404.200, Düren, Germany). The extracted RNA was dissolved in RNase-free water to obtain a concentration of 2 µg/µL. cDNA synthesis was performed using a RevertAid First Strand cDNA Synthesis Kit (K1621, Vilnius, Lithuania) according to the manufacturer’s instructions.

#### 2.3.2. Real-Time PCR Amplification

A Biorad CFX 96 Real-Time PCR Detection System (185-5096, Bio-Rad, USA) was used to measure the expression levels of three genes: FOXP3, RORC, and TBX21. Maxima SYBR Green/ROX qPCR Master Mix (2X) (K0221, Thermo Scientific) and gene-specific primers were used for the amplification. The reaction mix had 20 µL of nuclease-free water, 0.5 µL of each gene-specific primer, 2 µL of cDNA template, and 10 µL of 2X Maxima SYBR Green/ROX qPCR Master Mix. The PCR cycling conditions involved initial denaturation at 95 °C for 10 min, followed by 45 cycles of denaturation at 95 °C for 15 s, primer annealing at 60 °C for 40 s, and elongation at 72 °C for 40 s. The specificity of the amplified products was confirmed by melting curve analysis.

The Glyceraldehyde 3-phosphate dehydrogenase (*GAPDH*) gene was used as the reference gene to normalize the expression levels of the target genes. The expression levels of the target genes (*FOXP3*, RORγt (*RORC*), Tbet (*TBX21*)) were quantified relative to the expression of the housekeeping gene using the comparative Ct (2^−ΔΔCt^) method. The Ct values were converted to relative expression values using a formula that compares the target gene’s Ct value to the housekeeping gene’s Ct value. The relative expression values were then converted to Log2 values using the formula Log2 (relative expression). To calculate the Th1/Treg and Th17/Treg ratios, we divided the relative normalized mRNA expression of the gene RORγt (or T-bet) by the expression of FOXP3.

### 2.4. Gut Microbiota Analysis

#### 2.4.1. Firmicutes/Bacteroidetes Ratio

Participants were asked to provide a single stool sample, gathered in a sterile container, and promptly frozen at −80 °C for subsequent procedures. Using DNAzol by the manufacturer’s guidelines, DNA was extracted from a 100 mg portion of the frozen specimens [2]. The obtained DNA was dissolved in 200 μL of elution buffer, achieving the final DNA concentration for each sample. The 16 S rRNA gene was amplified through PCR using the CFX96 Touch Real-Time PCR Detection (185-5096, Bio-Rad, USA). The thermal cycling parameters were initiated with a 5 min denaturation at 95 °C, followed by 30 cycles involving 15 s at 95 °C for denaturation, 15 s at 61.5 °C for annealing, and 30 s at 72 °C for extension. A final extension step was performed at 72 °C for 5 min. Each PCR reaction included 0.05 units/μL of Taq polymerase (9012-90-2, Sigma Aldrich), 0.2 mM of each dNTP, 0.4 μM of each primer, 1x buffer, around 10 ng of DNA, and water to reach a final volume of 25 μL. All samples were subjected to triplicate amplification using the specified primer pairs. The thermal cycler recorded the threshold cycles (Cts) for general and specific primers.

In order to assess the makeup of gut microbiota at the phylum level, we utilized quantitative real-time PCR (qRT-PCR) with universal primers designed for the bacterial 16 S rRNA gene, as well as specific primers that target Firmicutes and Bacteroidetes. The threshold cycles (Cts) obtained from qRT-PCR were used to calculate the proportion of taxon-specific 16 S rRNA gene copies in each sample using the formula X = (Efficiency of Universal primers) ^Ct of Universal primers^/(Efficiency of Specific primers) ^Ct of Specific primers^ × 100 [27]. The efficiency of the universal primers was quantified as Efficiency of Universal primers, with a value of 2 indicating 100% efficiency and a value of 1 indicating 0% efficiency. The efficiency of the taxon-specific primers was denoted as Efficiency of Specific primers. The PCR amplification efficiency was assessed by performing a series of dilutions, and the fluorescence emitted by the SYBR Green dye was utilized to detect the amplified products. If the taxon-specific copy number in a sample was not detectable, it was assigned a value of 0. X represents the proportion of taxon-specific 16 S rRNA gene copies in a given sample, as indicated by the given equation.

Assessment of the gut microbial composition at the level of major phyla was carried out using quantitative real-time PCR (qRT-PCR) with the use of universal primers targeting the bacterial 16 S rRNA gene and specific primers for Firmicutes and Bacteroidetes (Table 1).

#### 2.4.2. Alpha-Diversity Indices Calculations

For the study of gut microbiota, a 1.0 g feces sample was obtained, and 9 mL of isotonic (0.9%) sodium chloride solution was put into a test tube. Through thorough mixing, a homogeneous mass was achieved, establishing a 10^−1^ dilution. Successive dilutions from 10^−2^ to 10^−11^ were prepared similarly. With clean micropipettes, 10 μL was taken from each dilution and put on nutrient media to separate certain microorganisms. Different types of commercial nutrient media made it easier to separate enterobacteria, yeast (*Candida* spp.), *Clostridium* spp., *Lactobacillus* spp., *Bifidobacterium* spp., and *Bacteroides* spp. Microorganism identification followed the guidelines outlined in the *Clinical Microbiology Procedures Handbook*, Volume 1–3, 4th Edition [28]. Decimal logarithms (lg CFU/g) were adopted for ease of presentation and subsequent mathematical and statistical processing of colony growth indicators.

To assess the alpha-diversity of the gut microbiota, the Shannon H′ and Simpson 1/D indices were employed. The Shannon H′ index was computed using the formula H′ = −∑ pi ln(pi), where pi denotes the proportion of individuals associated with each genus in the gut microbiota. The Simpson 1/D index was determined using the formula 1/D = ∑ pi^2^, with pi representing the proportion of individuals attributed to each genus in the gut microbiota. For diversity calculations, Abundance Curve Calculator was utilized.

### 2.5. Statistical Analysis

The data were analyzed using the statistical software R (version 4.3.1) and its associated packages, including tidyverse (version 1.3.0) and ggplot2 (version 3.4.4) for data manipulation and visualization. Additionally, the analysis was performed using GraphPad Prism (version 9) for graphical representation of the results of Th17/Treg and Th1/Treg ratios.

Descriptive statistics including mean and standard deviation (SD) were calculated for all variables. The normality of the data distribution was tested using the Shapiro–Wilk test. As the data were not normally distributed, non-parametric statistical tests were used for further analysis. To investigate the correlations between clinical data (NLR, CRP, PCT, monocytes), alpha-diversity indices, and Th17/Treg ratio, we conducted a correlation analysis using Spearman’s rank correlation coefficient depending on the normality of data distribution. All statistical tests were two-sided, and a *p*-value less than 0.05 was considered statistically significant. Additionally, we conducted a power analysis using a Sample Size Calculator to ensure adequate statistical power in our study [2].

## 3. Results

The average age of COVID-19 patients with T2D without metformin treatment (53.3% women and 47.7% men) was 54.88 ± 19.19 years, while those treated with metformin (60% men and 40% women) had an average age of 58.43 ± 6.27 years. However, the difference between these groups lacked statistical significance (*p* = 0.861).

### 3.1. Relative Expression of FOXP3, RORC, and TBX21 in Metformin-Treated COVID-19 Patients

**Figure 2:**
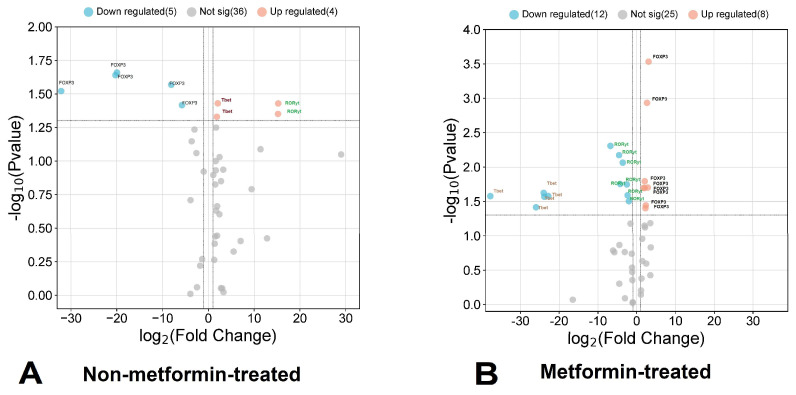
Volcano plot of the expression of investigated genes. The volcano plot vividly captures the transcriptional dynamics of key genes in fifteen COVID-19 patients who did not use metformin and fifteen COVID-19 patients who did use metformin. Five patients who did not take metformin had a significantly reduced expression of the *FOXP3* gene, and two more samples each had a significantly increased expression of *RORC* and *TBX21* (**A**). In contrast, increased expression of *FOXP3* and decreased expression of *RORC* and *TBX21* were observed in patients taking metformin (**B**).

In this study, we determined the mRNA expression of three key genes of T-helper subpopulations, i.e., FOXP3 (Treg), RORγt (Th17), and T-bet (Th1), in patients with COVID-19 and T2D who did or did not take metformin (Figure 2). To calculate the relative normalized expression using the PCR method, a control group of COVID-19 patients without T2D was used.

This study found that people who did not take metformin had a downregulated expression of FOXP3 by 6.62-fold, upregulated expression of RORγt by 29.0-fold, and upregulated T-bet by 1.78-fold, compared to the control group. In contrast, patients who took metformin showed a 1.96-fold upregulation of FOXP3, while RORt and T-bet showed 1.84-fold and 11.4-fold downregulations, respectively, compared to the control group.

### 3.2. Correlation between Th1/Treg, Th17/Treg mRNA Ratios, and Gut Microbiota Composition and Hematological Parameters

**Figure 3:**
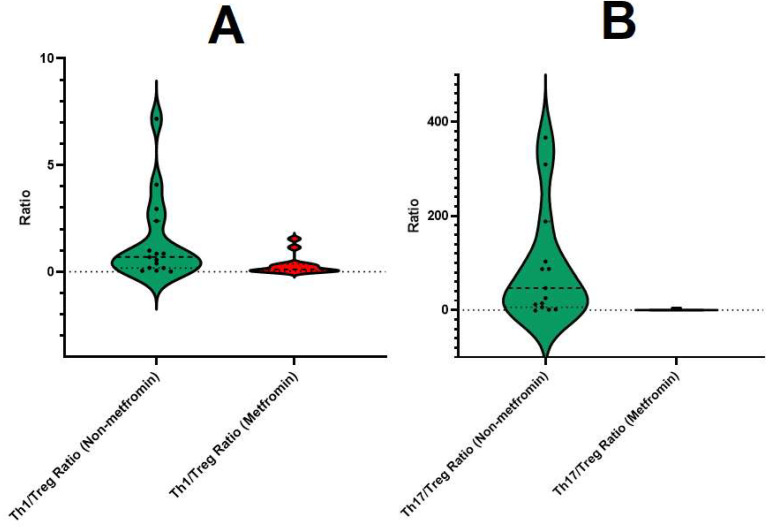
Violin plot of the distribution of Th1/Treg and Th17/Treg mRNA ratios among the investigated groups. Metformin-treated COVID-19 patients had reduced Th1/Treg (**A**) and Th17/Treg (**B**) relative expression ratios compared to metformin-treated COVID-19 patients.

We utilized the 2^−ΔΔCq^ (Livak) method for normalization to calculate the ratios of relative expression levels among three genes [29]. We calculated the ratio between the levels of relative normalized expression of the three genes and determined the peculiar ratios between them. Differences in the ratio between the groups that received or did not receive metformin were established. Patients taking metformin had lower levels of Th1/Treg (0.302 ± 0.44) compared to those who did not (1.42 ± 1.99) and lower Th17/Treg mRNA ratios (0.57 ± 1.17 vs. 149 ± 260) (Figure 3).

As for the gut microbiota, patients who did not take metformin had a significantly lower alpha-diversity measured by the Shannon and Simpson indices, but the F/B ratio did not differ significantly (1.5 ± 0.42 in the non-metformin-treated group vs. 1.09 ± 0.36 in the metformin-treated group).

Inflammatory markers in patients taking metformin, such as NLR (4.12 ± 0.88 vs. 14.53 ± 2.19), procalcitonin (0.32 ± 0.24 vs. 2.79 ± 0.87), and CRP (16.58 ± 2.28 vs. 27.38 ± 8.23), were significantly lower compared to patients not taking metformin.

**Figure 4:**
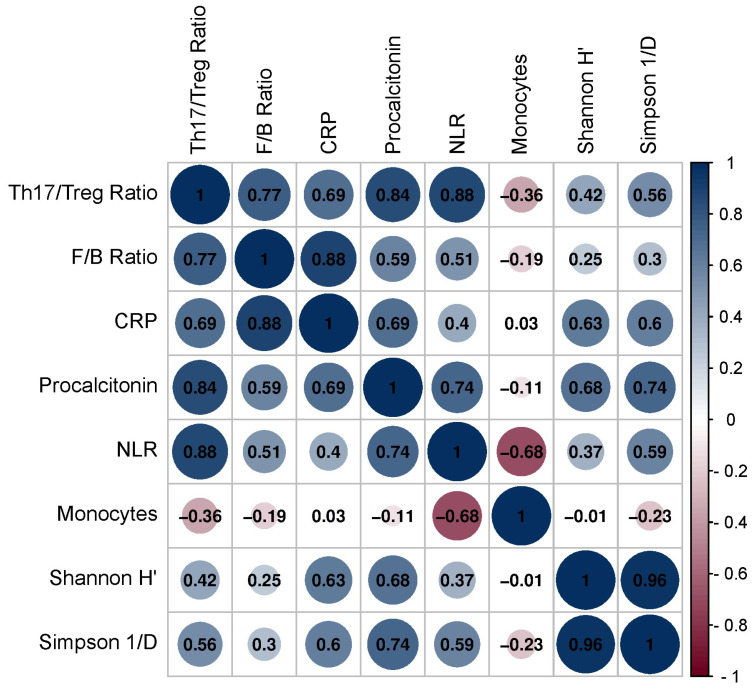
Correlation matrix between Th17/Treg ratio, gut microbiota, and inflammation markers. Positive associations were found between Th17/Treg ratio and Firmicutes/Bacteroidetes (F/B) ratio (r = 0.771, *p* = 0.042), procalcitonin (r = 0.844, *p* = 0.016), and Neutrophil-to-Lymphocyte ratio (NLR) (r = 0.878, *p* = 0.009).

Additionally, we conducted correlation analyses to explore potential relationships between inflammatory biomarkers, gut microbiota, and Th1/Treg and Th17/Treg mRNA ratios (Figure 4). A positive correlation was identified between the Th17/Treg mRNA ratio and the F/B ratio (r = 0.771, *p* = 0.042), procalcitonin (r = 0.844, *p* = 0.016), and NLR (r = 0.878, *p* = 0.009). A positive correlation was also observed between the F/B ratio and CRP (r = 0.815, *p* = 0.008).

## 4. Discussion

In this study, we aimed to investigate the potential relationships between inflammatory biomarkers, gut microbiota, and Th1/Treg and Th17/Treg ratios in individuals taking metformin. Our findings revealed a positive correlation between the Th17/Treg ratio and the F/B ratio, suggesting that an imbalance in the gut microbiota composition may contribute to the dysregulation of T cell subsets in these individuals. Furthermore, we observed positive correlations between the Th17/Treg ratio and procalcitonin, as well as the NLR, indicating that increased inflammation may be associated with an imbalance in T cell subsets.

Although we did not observe changes in the F/B ratio between patients taking and not taking metformin, several studies have reported that obese and diabetic patients exhibit a higher F/B ratio compared to healthy subjects [30,31]. Patients taking metformin showed higher indices of alpha diversity, aligning with findings from previous studies [32].

Metformin changes the balance of pro-inflammatory and anti-inflammatory T-lymphocytes by changing the way lymphocytes use energy by changing AMP-activated protein kinase (AMPK) [33,34]. Our earlier investigations revealed that individuals taking metformin exhibited elevated expression levels of the *PRKAA1* gene and reduced expression levels of *SLC2A1* and *MTOR* [35]. These molecular changes were correlated with decreased inflammatory markers. The current discovery serves as a substantial complement to our prior studies, providing valuable insights and enhancing our understanding of the favorable effects of metformin on the clinical outcomes of COVID-19. These findings suggest that metformin may have a direct impact on the immune response to viral infections, including COVID-19. By upregulating the expression of *PRKAA1* and downregulating *SLC2A1* and *MTOR*, metformin could promote the activation of anti-inflammatory T-lymphocytes and inhibit the production of inflammatory markers. This could potentially explain why individuals taking metformin have shown improved clinical outcomes when infected with COVID-19, as the drug may help mitigate the excessive immune response and cytokine storm often associated with severe cases of the disease.

In a trial by Ventura-López et al., metformin treatment exhibited significant benefits in comparison to the placebo group. Participants receiving metformin showed a notable decrease in the requirement for supplemental oxygen, a more marked reduction in the percentage of viral load, and a faster achievement of an undetectable viral load. Nevertheless, there were no noteworthy distinctions in the duration of hospitalization between the metformin-treated individuals and those in the placebo group [36]. In the TOGETHER Trial, the use of metformin did not yield a significant decrease in hospitalizations that was due to COVID-19. However, when considering the per-protocol sample, which accounted for 83% of the participants, there was a reduced likelihood of emergency department visits and hospitalizations of COVID-19 patients, resulting in an absolute risk reduction of 1.4% and 3.1%, respectively [37].

## 5. Conclusions

In conclusion, this study suggests that metformin may exert beneficial effects in COVID-19 patients with type 2 diabetes by influencing key immune response genes and modulating the gut microbiota. The upregulation of FOXP3 and downregulation of RORγt and T-bet point towards a potential anti-inflammatory impact, aligning with lower inflammatory markers observed in metformin-treated individuals. However, this study’s limitations, including a small sample size and the reliance on culture-based methods for microbiota analysis, call for cautious interpretation. Future research employing advanced sequencing techniques and larger cohorts is essential for a more comprehensive understanding. These findings hint at metformin’s potential role in immune modulation during COVID-19, but further well-controlled studies are needed to validate and refine these observations for clinical applications.

## 6. Limitation

1. Sample size and homogeneity. The sample size in this study may limit the generalizability of the results. A larger and more diverse cohort would strengthen the statistical power. The study participants were recruited from a single medical center, which may affect the representativeness of the findings. A multi-center approach involving different geographic locations and demographic groups could enhance the external validity of the results.
2. Confounders and co-morbidities. The presence of confounding factors and co-morbidities may influence the observed associations. While efforts were made to exclude participants with chronic diseases and other gastrointestinal disorders, the influence of uncontrolled confounders cannot be entirely ruled out. This study did not systematically control for the influence of obesity and dietary habits on the gut microbiota and inflammatory markers. Both obesity and diet are known to be crucial factors influencing microbiome diversity and immune responses.
3. Gut microbiota analysis. This study employed a culture-based method for calculating alpha-diversity indices, providing insights into the relative abundance of specific bacterial taxa. However, it is crucial to acknowledge that culture-based methods have limitations in capturing the entire spectrum of microbial diversity present in the gut.
4. Cross-sectional design. The cross-sectional design of this study limits the establishment of causal relationships. Longitudinal studies would provide a more dynamic understanding of the relationship between metformin use, gut microbiota, inflammatory markers, and gene expression levels over time.

## Figures and Tables

**Table 1:** Primer nucleotide sequences used for qRT-PCR assay.

Phylum	Primer Nucleotide Sequence	
	Forward	Reverse
Firmicutes	928F-firm TGAAACTYAAGGAATTGACG	1040FirmR ACCATGCACCACCTGTC
Bacteroidetes	798cfbF CRAACAGGATTAGATACCCT	cfb967R GGTAAGGTTCCTCGCGCTAT
16S rRNA gene	926F AAACTCAAAKGAATTGACGG	1062R CTCACRRCACGAGCTGAC

## Data Availability

The data presented in this study are available on request from the corresponding author.
